# Comparative Effects of Selenium Yeast and Sodium Selenite on the Selenium Distribution, Interior Quality and Oxidative Stability of Docosahexaenoic Acid-Enriched Eggs During Storage

**DOI:** 10.3390/antiox14111333

**Published:** 2025-11-05

**Authors:** Chenhao Zou, Chaoyue Ge, Yujie Lv, Weichen Huang, Shenao Zhan, Xinyu Shen, Hongmeng Yuan, Xiaoxu Wang, Lianchi Wu, Dongyou Yu, Bing Liu

**Affiliations:** 1 College of Animal Sciences, Zhejiang University, Hangzhou 310058, China; chenhao.zou@zju.edu.cn (C.Z.); 22117078@zju.edu.cn (C.G.); 22217093@zju.edu.cn (Y.L.); 22317098@zju.edu.cn (W.H.); 22317012@zju.edu.cn (S.Z.); 22317063@zju.edu.cn (X.S.); 22417094@zju.edu.cn (H.Y.); 22417088@zju.edu.cn (X.W.); 22217068@zju.edu.cn (L.W.); 2 Hainan Institute, Zhejiang University, Sanya 572025, China; 3 ZJU-Xinchang Joint Innovation Centre (TianMu Laboratory), Gaochuang Hi-Tech Park, Shaoxing 312500, China

**Keywords:** selenium source, interior quality, storage stability, oxidative stability, egg

## Abstract

Docosahexaenoic acid (DHA)-enriched eggs are nutritionally valuable for human cardiovascular health and neurodevelopment but face severe lipid oxidation during storage due to DHA’s high degree of unsaturation, which reduces their quality and shelf life. Selenium (Se) mitigates such oxidation, yet the efficacy of different Se sources (organic vs. inorganic) in DHA-enriched eggs remains inadequately quantified. This study investigated the effects of dietary Se sources on Se distribution, internal quality, and oxidative stability of DHA-enriched eggs by feeding 360 Hy-line Brown laying hens (50 weeks old) four diets for 8 weeks: a basal diet (CON; analyzed Se: 0.10 mg/kg), a DHA-enriched microalgae diet (MA; analyzed Se: 0.11 mg/kg), or MA supplemented with 0.25 mg/kg Se as sodium selenite (MA + SS) or selenium yeast (MA + SY). At the end of the feeding trial, eggs were collected and stored at 22 °C for 0, 15, or 30 days to evaluate internal quality and oxidative stability. Results showed that SY was significantly more effective than SS in enriching Se in eggs: the total Se content in whole eggs of MA + SY (18.82 mg) was 39.6% higher than that of MA + SS (13.48 mg), with albumen Se content in MA + SY (0.239 mg/kg) being 2.17-fold that of MA + SS (0.110 mg/kg). Supplementation with DHA alone (MA diet) negatively impacted stored egg quality: at 30 days of storage, the Haugh unit (HU) of MA (54.93) was 10.6% lower than that of CON (61.48), and yolk thiobarbituric acid-reactive substances (TBARSs, 495.8 μg MDA/kg) was 22.9% higher than that of CON (403.3 μg MDA/kg). However, both Se sources improved these parameters, with SY showing a more pronounced effect: at 30 days, MA + SY had a higher yolk GPX activity (58.10 U/g protein, 12.1% higher than MA + SS) and lower yolk TBARSs (361.2 μg MDA/kg, 11.6% lower than MA + SS), while its HU (62.97) was restored to 99.2% of CON’s level. The superior efficacy of SY was attributed to greater Se deposition and enhanced GPX activity, which jointly reduced lipid and protein oxidation. These findings confirm SY as the preferred Se supplement for producing nutritionally enhanced DHA-enriched eggs with improved storage stability.

## 1. Introduction

Long-chain n-3 polyunsaturated fatty acid (n-3 LC-PUFA)-enriched foods have gained increasing popularity due to their well-documented benefits in cardiovascular disease prevention and neurodevelopmental support [1,2]. Eggs, as a globally accessible and affordable food commodity, represent one of the most effective vehicles for delivering n-3 LC-PUFAs into human diets. Microalgae, recognized as a primary natural source of n-3 LC-PUFAs, have emerged as a promising ingredient for producing n-3 PUFA-enriched eggs, offering advantages in enrichment efficiency, production sustainability, and egg sensory quality [3]. A recent meta-analysis confirmed that microalgae supplementation improves egg quality parameters (increased fatty acid content, β-carotene concentration, and yolk color intensity) without negative affects laying performance. Notably, brown microalgae demonstrate greater efficacy than green varieties in enriching n-3 fatty acids [4,5].

However, n-3 LC-PUFA enrichment increases lipid unsaturation in egg yolks, thereby enhancing susceptibility to oxidation [6,7]. Lipid peroxidation accelerates the deterioration of nutritional and sensory qualities, shortens shelf life, and produces oxidation products associated with increased risks of fatty liver and inflammatory diseases [8,9]. Additionally, lipid peroxidation negatively impacts albumen’s physicochemical and functional properties through oxidative modifications [10]. These oxidative phenomena are exacerbated during storage, particularly at elevated temperatures, and are further accelerated by domestic cooking processes such as frying and boiling [11]. Consequently, various antioxidants have been incorporated into hen diets to mitigate oxidation and improve consumer acceptance of n-3 PUFA-enriched eggs [12]. Selenium (Se), an essential micronutrient for avian species, exerts antioxidant effects against lipid and protein oxidation through regulation of Se-dependent selenoenzymes, including glutathione peroxidase (GPX) which is present in both albumen and yolk [13,14]. Dietary Se modification to enhance GPX activity may therefore preserve egg freshness while simultaneously providing a promising strategy to meet daily Se requirements through egg consumption. Historically, sodium selenite (SS) has been widely used in poultry diets, but its application is constrained by acute toxicity concerns [15].

Selenium yeast (SY), produced via aerobic fermentation, has demonstrated superior bioactivity, lower toxicity, and enhanced tissue accumulation compared to sodium selenite (SS), leading to its increasing advocacy and approval in recent years [15,16]. Numerous studies have confirmed that 0.3 mg/kg SY outperforms SS in improving laying performance, tissue Se deposition, antioxidant capacity, and immune response in laying hens [17]. SY’s high efficiency in egg Se enrichment is attributed to its high content of selenomethionine, which can be incorporated into proteins through methionine metabolic pathways, resulting in significantly higher Se concentrations in eggs compared to SS [13,18]. Despite these advances, limited research has compared the effects of dietary Se sources on the internal quality and oxidative stability of stored eggs, particularly in the context of n-3 PUFA-enriched eggs. Therefore, this study aimed to investigate the effects of 0.25 mg/kg dietary Se supplementation from either SS or SY on Se distribution, internal quality (freshness), and oxidative stability of eggs from laying hens fed docosahexaenoic acid (DHA)-enriched microalgae diets.

## 2. Materials and Methods

### 2.1. Materials

The 1.5% DHA-enriched microalgae (MA) and sodium selenite (SS) were purchased from Alltech Inc. (Nicholasville, KY, USA), while selenium yeast (SY) was obtained from Tiandichun Chemical Reagent Co., Ltd. (Weifang, Shandong, China).

### 2.2. Experimental Design and Diet

The experimental use of animals and procedures was approved by the Institutional Animal Care and Use Committee of Zhejiang University (Protocol number: ZJU20220310). A total of 360 Hy-Line Brown laying hens (50 weeks of age) were randomly assigned to four dietary treatments. Each dietary treatment included 6 replicates, with each replicate consisting of a group of 15 hens housed in 5 adjacent cages (40 × 40 × 45 cm per cage, 3 hens per cage). All birds were acclimated to a basal diet for 2 weeks. At the end of week 52, the birds were assigned to one of four dietary treatments: CON, the basal diet with soybean oil without Se supplementation; MA, the basal diet supplemented with 1.5% DHA-enriched microalgae without selenium supplementation; MA + SS and MA + SY, the MA diets further supplemented with 0.25 mg Se/kg diet from SS (4560 mg Se/kg content, Tiandichun Chemical Reagent Co., Ltd., Weifang, Shandong, China) or SY (a kind of seleno-yeast product Sel-Plex^®^, 1000 mg Se/kg content, Alltech Co., Ltd. Nicholasville, KY, USA), respectively. All Se supplements were incorporated into a premix before being used in the production of the complete feed. The experimental diets were formulated in accordance with the China Agricultural Standard [19] and the NRC [20] guidelines to meet the nutrient requirements of laying hens (Table 1). All diets were designed to be isocaloric and isonitrogenous. The measured Se concentrations in the basal and experimental diets are presented in Table 2. Birds had ad libitum access to feed and water and were housed in environmentally controlled rooms maintained at a constant temperature of 23 ± 2 °C and a relative humidity of 60–70%, under a 16:8 h light-dark cycle [21].

### 2.3. Blood and Tissue Collection

After 8 weeks of experimentation, 1 hen close to the average weight of each replicate group was selected per treatment (6 hens total, corresponding to 6 unites), and serum/organ samples from each selected hen were attributed to its respective unit. The collected blood samples were then centrifuged at 3000× *g* for 20 min [22], the supernatant (serum) were obtained for total Se analysis. Then the selected hens were euthanized humanely by cervical dislocation, and the heart, liver, spleen, and kidney were collected after exsanguination for total Se analysis.

### 2.4. Egg Sample Collection and Preparation

After 8 weeks of experimentation, 5 eggs/replicate (a total of 30 fresh eggs per treatment) were randomly selected for analysis of Se, docosahexaenoic acid (DHA), and chemical composition. For egg detection, 5 eggs were pooled to form 1 detection unit, each treatment was set with 6 detection units (corresponding to a total of 30 eggs per treatment), which served as the experimental units for the statistical analysis of egg-related indicators. Yolks from separated from the albumen for determination of Se, total lipid, cholesterol, DHA, and other chemical components, while the albumen was also pooled for analysis of Se content and chemical composition. Another 360 fresh eggs (90 eggs per treatment) were randomly selected, labeled, and then stored at 22 °C (room temperature) for 0, 15, and 30 days to evaluate egg freshness and oxidative stability. On each scheduled day, egg weight and internal quality traits. The yolk index was calculated as the ratio of yolk height to yolk diameter. The yolks were pooled per replicate for subsequent analysis of yolk pH, glutathione peroxidase (GPX) activity, and thiobarbituric acid-reactive substances (TBARSs). Similarly, the corresponding albumens were pooled per unite to evaluate GPX activity.

### 2.5. Se Concentration in Feed, Serum, Tissue, and Egg

Se concentrations were determined following the method described by us previously [23]: for sample pretreatment, feed or tissue (heart, liver, spleen, kidney) samples were prepared by weighing 0.5 g of homogenized sample into a polytetrafluoroethylene digestion vessel, adding 5 mL of a nitric acid-perchloric acid mixture (4:1, *v*/*v*), incubating overnight at room temperature, digesting via a microwave digester (Mars 6, CEM Corporation, Matthews, NC, USA) with the program; egg albumen or yolk samples were prepared by pooling albumen or yolk from 5 eggs per replicate, homogenizing, weighing 1.0 g for digestion (following the same procedure as tissue samples), and diluting to 20 mL with 5% hydrochloric acid. Atomic fluorescence spectrophotometry was performed using an AFS-930 atomic fluorescence spectrometer (Beijing Jitian Instrument Co., Ltd., Beijing, China) with the following parameters: Se hollow cathode lamp current 60 mA, negative high voltage 270 V, carrier gas (Ar) flow rate 400 mL/min, shielding gas flow rate 800 mL/min, and reaction temperature 200 °C. For quantification, a Se standard solution was used to prepare a standard curve (0.0, 1.0, 2.0, 5.0, 10.0 μg/L), and the recovery rate of Se in spiked samples (5 μg/kg) ranged from 95.2% to 102.3% with a relative standard deviation (RSD) < 5%.

### 2.6. Chemical Composition Analysis

Dry matter, ash, and crude proteins in the yolk and albumen were determined in accordance with the Association of Official Analytical Chemists [24]. For dry matter, 1.0 g of homogenized sample was dried in an electric thermostatic drying oven (DHG-9070A, Shanghai Yiheng Scientific Instrument Co., Ltd., Shanghai, China) at 105 °C to constant weight, and the content was calculated based on weight loss. For ash, 2.0 g of sample was incinerated in a muffle furnace (SX2-4-10N, Shanghai Yiheng Scientific Instrument Co., Ltd., Shanghai, China) at 550 °C for 6 h until white ash was obtained. For crude protein, the Kjeldahl method was adopted: 0.5 g of sample was digested with concentrated sulfuric acid and a catalyst (CuSO_4_-K_2_SO_4_, 1:10, *w*/*w*) in a digestion furnace (Kjeltec 2300, FOSS, Hillerød, Denmark), followed by distillation (Kjeltec 8400, FOSS, Hillerød, Denmark) and titration with 0.1 mol/L hydrochloric acid.

### 2.7. Egg Freshness Quality Traits

Egg freshness quality traits (egg weight, albumen height, and HU) were assessed using a digital tester (DET6000, Nabel Co., Ltd., Osaka, Japan) on five eggs per replication. Weight loss percentage after 15 or 30 days of storage was calculated relative to the initial egg weight on day 0. The yolk index was determined as the ratio of yolk height to yolk width, measured with a digital caliper. Yolk pH values were measured using a calibrated pH meter (HI99161, Hanna, Rome, Italy). Total lipid content was measured via the Soxhlet extraction method with a Soxhlet extractor (SZF-06A, Shanghai Xinjia Instrument Co., Ltd., Shanghai, China). Cholesterol levels were analyzed using a commercial assay kit (Catalog No. A111-1-1, Nanjing Jiancheng Bioengineering Institute, Nanjing, China) in conjunction with a microplate reader (Infinite M200 Pro, Tecan Group Ltd., Männedorf, Switzerland) [25]. Atomic fluorescence spectrophotometry was performed using an AFS-930 atomic fluorescence spectrometer (Beijing Jitian Instrument Co., Ltd., Beijing, China) with the following parameters: Se hollow cathode lamp current 60 mA, negative high voltage 270 V, carrier gas (Ar) flow rate 400 mL/min, shielding gas flow rate 800 mL/min, and reaction temperature 200 °C.

### 2.8. Determination of GPX Activity, Lipid and Protein Oxidation

The activity of GPX was determined according to the method described by Miao et al. [26], using a commercial GPX assay kit (Catalog No. A005, Nanjing Jiancheng Bioengineering Institute, Nanjing, China). Lipid oxidation in yolk was assessed following the procedure of Galobart [27]: 2.0 g of yolk was mixed with 8 mL of 7.5% trichloroacetic acid containing 0.1% EDTA, vortexed for 2 min, centrifuged at 4000× *g*, 2 mL of filtrate was reacted with 2 mL of 0.02 M thiobarbituric acid in a boiling water bath for 30 min, and absorbance at 532 nm was measured using a spectrophotometer; TBARS values were derived from a standard curve prepared with malondialdehyde (MDA) tetrabutylammonium salt (Sigma-Aldrich, St. Louis, MO, USA) and expressed as mg MDA/kg fresh egg yolk. Protein oxidation in albumen was evaluated by Bayarsaikhan et al. [28]: 1.0 mL of albumen was diluted with 4 mL of 0.2 M PBS, mixed with 1.0 mL of 10 mM DNPH, and incubated in the dark at room temperature for 1 h. Then, 1.0 mL of 20% TCA was added to precipitate proteins, followed by centrifugation at 3000× *g*. Subsequently, the pellet was dissolved in 2 mL of 6 M guanidine hydrochloride, and the absorbance at 370 nm was measured using a spectrophotometer. Carbonyl content was calculated using a molar extinction coefficient of 22,000 M^−1^ cm^−1^ and expressed as nmol/mg protein.

### 2.9. Statistical Analysis

Data obtained from 6 units per dietary treatment (expressed as mean ± standard deviation, SD) were analyzed using the PROC GLM procedure of SPSS 23.0 software (SPSS Inc., Chicago, IL, USA) to perform analysis of variance (ANOVA) according to the method described by Lv [29]. For serum and organ indicators, one-way analysis of variance (ANOVA) was performed. For egg storage-related indicators (e.g., Haugh unit, yolk index, TBARS value), two-way ANOVA was applied to evaluate the main effects of dietary treatment, storage time, and their interaction. Significant differences (*p* < 0.05) among dietary treatments were identified by Tukey’s multiple comparisons test. The correlations between variables were determined by Pearson’s correlation coefficient analysis, and the correlation results were visualized using heatmaps generated with R 4.2.2 software (R Foundation for Statistical Computing, Vienna, Austria) and the ‘pheatmap’ package.

## 3. Results and Discussion

### 3.1. Actual Se Content of the Experimental Diet

The actual selenium (Se) content of the four experimental diets is presented in Table 2, with their formulated and analyzed values showing good consistency: the analyzed Se levels were 0.10 mg/kg for the control diet (CON) and 0.11 mg/kg for the 1.5% DHA-enriched microalgae-supplemented diet (MA), matching their respective formulated values of 0.11 and 0.12 mg/kg; for the Se-supplemented groups, the analyzed Se contents of the MA diet plus sodium selenite (MA + SS) and MA diet plus selenium yeast (MA + SY) were 0.36 and 0.35 mg/kg, which were close to their formulated values of 0.36 and 0.37 mg/kg. All diets were formulated to be isocaloric and isonitrogenous in accordance with the China Agricultural Standard [19] and NRC [20] guidelines (Table 1), ensuring no variations in energy or protein content across treatments. This consistency in Se content is foundational to the study’s validity, as tissue and egg Se deposition are directly dependent on actual dietary Se dosage [30,31].

### 3.2. Selenium Contents in Serum and Tissues

Dietary supplementation with 0.25 mg/kg Se (in the MA + SS and MA + SY groups) significantly increased (*p* < 0.05) Se deposition in serum and various tissues compared to diets (CON and MA groups) in laying hens (Table 3). The CON and MA groups had the lowest serum Se concentrations, with no significant difference between them (*p* > 0.05), whereas the MA + SY group exhibited a significantly higher (*p* < 0.05) serum Se level than the MA + SS group, with an increase of 11.4%. In the liver, Se deposition in the MA + SY group was significantly greater (*p* < 0.05) than that in the MA + SS group, showing an 8.3% increase. However, no significant differences (*p* > 0.05) in Se content were observed between the MA + SS and MA + SY groups in the kidney, spleen, or heart. These results demonstrate that SY promotes Se deposition in serum and liver more effectively than SS in laying hens. These findings align with established knowledge that SY possess higher bioavailability in poultry than SS [32,33]. SY is rich in selenomethionine (SeMet), which is integrated into the methionine metabolic pathway—enabling active transport and deposition in tissues. In contrast, SS relies on passive absorption and is more prone to excretion, limiting its tissue accumulation [34,35]. The tissue-specific superiority of SY in serum and liver reflects the metabolic roles of these organs. This makes serum and liver more sensitive to differences in Se bioavailability, explaining why SY’s advantage is most pronounced here. The lack of difference in the kidney, spleen, and heart likely stems from their lower metabolic demand for Se or inherent capacity to maintain Se homeostasis, further emphasizing that SY’s benefits are most impactful in tissues critical for Se utilization and subsequent transfer to eggs.

### 3.3. Selenium Concentration and Total Selenium Amount in Eggs

Supplemental Se in hen diets (MA + SS and MA + SY groups) significantly increased (*p* < 0.05) Se content in both yolk and albumen compared to those from hens fed diets without Se supplementation (CON and MA groups; Table 4). No significant differences (*p* > 0.05) were observed in yolk Se content between the MA + SY and MA + SS treatments. However, albumen Se content was significantly higher (*p* < 0.05) in eggs from hens fed SY relative to those fed an equivalent dose of SS: the MA + SY group had 2.17-fold higher albumen Se than the MA + SS group. For total Se content in the whole egg (sum of albumen and yolk Se, excluding eggshell), the MA + SY group exhibited a significantly higher level than MA + SS group, with an increase of 39.6%, indicating that SY promotes greater Se accumulation in eggs than SS, particularly in the albumen. This distribution pattern is attributed to the chemical form of Se in each source. SY is incorporated into albumen proteins via methionine transporters, which do not discriminate between methionine and SeMet—allowing active integration into proteins synthesized in the hen’s oviduct for albumen formation [36]. In contrast, SS provides inorganic Se, which cannot be integrated into proteins and instead accumulates in the lipid-rich yolk via passive diffusion [37]. This distinction holds significant nutritional relevance for consumers: the albumen is a major source of high-quality, bioavailable protein, and higher Se content in MA + SY albumen means consumers obtain Se alongside protein. As previously reported, organic Se sources are more effective for egg Se biofortification [17,18], and the current data further support SY’s role as a superior supplement for producing Se-enriched, nutritionally enhanced eggs. Additionally, the higher total Se in MA + SY eggs better contributes to meeting human daily Se requirements, a key objective of Se-biofortified food production.

### 3.4. Chemical Composition of Eggs

Except for docosahexaenoic acid (DHA) in yolk, the four groups had no significant (*p* > 0.05) differences in other chemical components of yolk (dry matter, ash, total lipid, protein, total cholesterol) and albumen (dry matter, ash, protein; Table 5). Compared with the CON group, the MA group exhibited a significantly higher (*p* < 0.05) yolk DHA content: the MA group had 3.37-fold higher DHA than the CON group. The MA + SY group exhibited a significantly higher yolk DHA concentration compared to the MA + SS group (*p* < 0.05), with a 5.0% increase observed in the former. In contrast, MA + SS group did not differ significantly (*p* > 0.05) from MA, indicating that SY may enhance DHA accumulation in egg yolk more effectively than SS. The absence of differences in other chemical components is practically relevant, as it ensures Se supplementation does not compromise the sensory or nutritional attributes consumers expect from eggs (e.g., protein content, cholesterol levels)—maintaining consumer acceptance of DHA-enriched eggs [5]. The higher DHA content in MA + SY eggs is likely explained by SY’s antioxidant properties. By enhancing Se deposition and antioxidant enzyme activity, SY protects DHA from oxidation during yolk formation—reducing DHA loss and increasing its final concentration. This advantage is particularly important for DHA-enriched eggs. Since consumers focus on the DHA content of these products, SY’s ability to protect and enhance DHA levels further elevates their nutritional value [38].

### 3.5. Oxidative Stability of Eggs During Storage

Dietary SY supplementation significantly increased (*p* < 0.05) the activity of GPX in both the yolk and the albumen, as well as notably decreased (*p* < 0.05) the yolk TBARS values of fresh eggs compared with the CON group (Table 6). At 0 days, the MA + SY group had 1.32-fold higher albumen GPX activity than the CON group, and its yolk TBARS value was 12.5% lower than that of the CON group. Both dietary treatments and storage time significantly affected (*p* < 0.05) GPX activity, TBARS values, and protein carbonyl groups in eggs during storage. In general, regardless of Se source, Se supplementation increased GPX activity in both yolk and egg white and decreased albumen carbonyl content and yolk TBARS values in stored eggs to varying degrees. For example, at day 30, the MA + SY group exhibited 1.50-fold higher yolk GPX activity than the MA group, and its albumen carbonyl content was 28.1% lower than that of the MA group. Although both the MA + SS and MA + SY groups exhibited increased oxidative damage with extended storage, the rate of oxidative deterioration in MA + SY was slower. The 30-day yolk TBARS value of MA + SY was 11.6% lower than MA + SS. Storage accelerates oxidation in DHA-enriched eggs due to DHA’s high unsaturation, which increases susceptibility to free radical attack, and ambient temperatures (22 °C), which enhance molecular motion and exacerbate oxidative reactions [39,40,41]. Se supplementation mitigates this by upregulating GPX, a Se-dependent antioxidant enzyme that scavenges lipid peroxides and prevents protein oxidation [42,43,44]. SY’s superiority over SS stems from two key factors: first, SY enhances Se deposition in eggs, providing more substrate for GPX synthesis; second, SeMet from SY is more efficiently integrated into functional GPX [45], resulting in higher enzyme activity than SS-derived Se [46]. This enhanced GPX activity directly reduces the accumulation of oxidative damage markers in MA + SY eggs, indicating that SY was more effective than SS in improving the oxidative stability of eggs during storage.

### 3.6. Egg Freshness During Storage

The CON and MA groups showed poor freshness retention during room temperature storage, with higher weight loss rate, lower albumen height and HU, higher yolk pH, and lower yolk index compared to the supplemental Se in hen diets (MA + SS and MA + SY groups); their freshness decreased significantly (*p* < 0.05) as storage time prolonged (Table 7). Storage-induced freshness loss is driven by three key processes: water evaporation (causing weight loss), CO_2_ loss (increasing yolk pH by reducing buffering capacity), and degradation of the ovomucin-lysozyme complex in albumen (reducing albumen height and HU) [47,48]. DHA enrichment (MA group) exacerbates these changes because oxidative damage to albumen proteins impairs their water-holding capacity and structural integrity [10]. Although the freshness of both Se-supplemented groups declined with storage time, MA + SY maintained better freshness parameters throughout the storage period. Significant differences (*p* < 0.05) were observed between MA + SY and MA + SS: at both 15 and 30 days of storage, MA + SY exhibited a significantly lower weight loss rate compared to MA + SS. At 30 days of storage, MA + SY exhibited superior freshness retention relative to MA + SS across multiple key parameters. Specifically, MA + SY had a weight loss rate that was 13.8% lower than MA + SS. Additionally, MA + SY demonstrated significantly higher albumen height and HU (*p* < 0.05), with its HU being 5.6% higher than that of MA + SS. Furthermore, MA + SY showed a significantly lower (*p* < 0.05) yolk pH (0.6% lower than MA + SS) and a significantly higher (*p* < 0.05) yolk index (4.8% greater than MA + SS) at this storage time point. Consistent with previous reports [49], our study observed increased weight loss and yolk pH, alongside decreased HU and albumen height during storage. Notably, these deteriorative changes were significantly attenuated in Se-supplemented groups, particularly with SY. This protective effect aligns with the established link between oxidative degradation and quality loss in stored eggs [50]. This demonstrates that SY not only improves the nutritional quality of DHA-enriched eggs but also extends their shelf life, enhancing their viability for commercial distribution.

### 3.7. Pearson Correlation Analysis Between Selenium Content and Key Egg Quality Indicators

**Figure 1:**
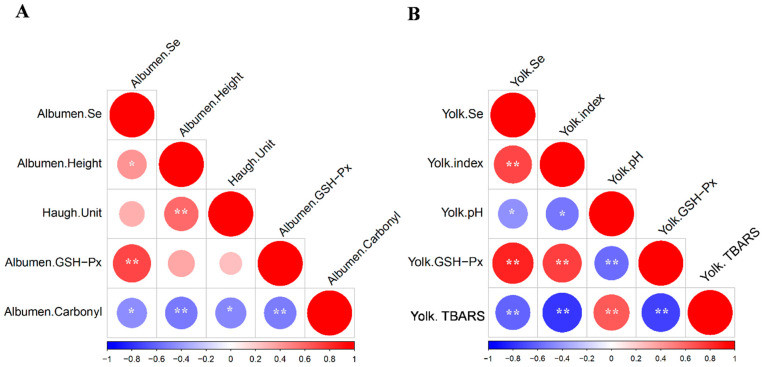
Pearson’s correlation coefficients between Se content, albumen height, Haugh unit, GPX and carbonyl groups in albumen (**A**), and Pearson’s correlation coefficients between Se content, yolk index, yolk pH, GPX and TBARS value of yolk (**B**). ** Correlation is significant at *p* < 0.01 and * correlation is significant at *p* < 0.05.

Pearson’s correlation analysis (Figure 1) revealed significant relationships between Se content and key egg quality indicators (*p* < 0.05): in albumen, albumen Se content was positively correlated with GPX activity (*p* < 0.05) and HU (*p* < 0.05), but negatively correlated with carbonyl group content (*p* < 0.05; Figure 1A); in yolk, yolk Se content was positively correlated with GPX activity (*p* < 0.05) and yolk index (*p* < 0.05), while it was negatively correlated with TBARS value (*p* < 0.05) and yolk pH (*p* < 0.05; Figure 1B), indicating that Se deposition plays a regulatory role in enhancing egg oxidative stability and maintaining freshness. These correlations validate the causal links between Se deposition, antioxidant capacity, and egg quality. The positive correlation between selenium (Se) and glutathione peroxidase (GPX) activity confirms that egg Se content directly enhances antioxidant capacity; higher Se levels promote greater GPX activity, thereby reducing oxidative damage [43,44]. The negative correlation between Se and oxidative markers (TBARSs, carbonyls) further reinforces that Se-mediated antioxidant activity mitigates oxidation. Meanwhile, the positive correlation between Se and freshness indicators (HU, yolk index) and negative correlation with yolk pH demonstrate that reduced oxidation directly translates to better freshness retention. Collectively, these correlations consolidate the study’s findings that SY enhances Se deposition, thereby increasing GPX activity, reducing oxidative stress, and preserving freshness, thus confirming SY’s superiority as a Se supplement for DHA-enriched eggs.

## 4. Conclusions

From a human nutritional health perspective, dietary supplementation with natural SY facilitates greater Se enrichment in eggs compared to SS, making SY a more effective choice for producing Se-biofortified eggs. Inclusion of DHA to the hen diets, in the absence of Se supplementation, adversely affects the interior quality and oxidative stability of stored eggs. Selenium supplementation effectively mitigates these adverse effects by enhancing the internal quality and oxidative stability of DHA-enriched eggs during storage, with SY demonstrating a more pronounced protective effect compared to SS. However, it should be noted that the synergistic effect between Se and other feed components (e.g., vitamin E) was not considered in this study, which may have led to an underestimation of Se’s antioxidant potential. From the perspective of industrial application, a key challenge is that the cost of SY is approximately 3–4 times that of SS; therefore, large-scale use requires optimizing the addition level to balance cost and efficacy. To further promote the industrial application of DHA-enriched eggs, future research could focus on combining compound antioxidants (e.g., vitamin E + Se) to maximize the antioxidant effect and exploring the response differences in different breeding varieties (e.g., white-shell laying hens) to Se sources. In summary, dietary SY supplementation not only achieves efficient Se biofortification of DHA-enriched eggs but also better alleviates DHA-induced oxidative deterioration during storage compared to SS, highlighting its potential in producing nutritionally enhanced (high DHA + high bioavailable Se) table eggs with improved storage stability.

## Figures and Tables

**Table 1:** Composition and nutrient levels of the basal control and microalgae diets (as fed basis, %).

Items	Control (CON)	Microalgae (MA)
Ingredients		
Corn	61.50	61.50
Soybean meal, 46% CP	25.00	24.50
Soybean oil	1.50	0.50
Microalgae	0	1.50
Limestone	8.50	8.50
CaHPO_4_	1.00	1.00
Salts	0.30	0.30
DL-Methionine	0.12	0.12
Lysine-HCl	0.08	0.08
V + M Premix ^1^	2.00	2.00
Total	100.00	100.00
Nutrient levels ^2^		
ME, Mcal/kg	2.73	2.72
CP, %	16.63 (16.57)	16.61 (16.58)
Lys, %	0.89	0.89
Met, %	0.38	0.38
Cys + Met, %	0.75	0.75
Ca, %	3.66 (3.70)	3.65 (3.69)
Total P, %	0.55	0.55
Av. P, %	0.35	0.35
Se, mg/kg	0.11 (0.10)	0.12 (0.11)

^1^ The V + M premix provided the following per kilogram of diet: VA, 6250 IU; VD_3_, 3125 IU; VE, 15 IU; VK, 2 mg; thiamine, 1 mg; riboflavin, 8.5 mg; calcium pantothenate, 50 mg; niacin, 32.5 mg; pyridoxine, 8 mg; folate, 5 mg; VB12, 125 μg; choline, 500 mg; Fe, 60 mg; Cu, 8 mg; Mn, 65 mg; Zn, 60 mg; and I, 1 mg. ^2^ The values in parentheses indicated the analyzed value. Others are calculated values.

**Table 2:** Se content (mg/kg) in the experimental diets.

Treatment	Se Sources	Supplemental Se (mg/kg)	Calculated Total Se (mg/kg)	Analyzed Total Se (mg/kg)
CON	-	0	0.11	0.100 ± 0.008
MA	-	0	0.12	0.110 ± 0.008
MA + SS	sodium selenite	0.25	0.36	0.360 ± 0.007
MA + SY	selenium yeast	0.25	0.37	0.350 ± 0.004

**Table 3:** Se content in serum (μg/mL) and tissues (μg/g fresh tissue) of laying hens fed DHA-enriched diets with sodium selenite or selenium yeast supplementation.

Items	CON	MA	MA + SS	MA + SY	*p*-Value
Serum	0.074 ± 0.009 ^c^	0.068 ± 0.008 ^c^	0.176 ± 0.013 ^b^	0.196 ± 0.009 ^a^	<0.001
Liver	0.258 ± 0.015 ^c^	0.219 ± 0.012 ^d^	0.458 ± 0.023 ^b^	0.496 ± 0.016 ^a^	<0.001
Kidney	0.408 ± 0.025 ^b^	0.420 ± 0.026 ^b^	0.627 ± 0.032 ^a^	0.603 ± 0.033 ^a^	<0.001
Spleen	0.403 ± 0.013 ^b^	0.406 ± 0.018 ^b^	0.575 ± 0.015 ^a^	0.583 ± 0.017 ^a^	<0.001
Heart	0.302 ± 0.021 ^b^	0.311 ± 0.019 ^b^	0.474 ± 0.021 ^a^	0.492 ± 0.011 ^a^	<0.001

Each value represents the mean of 6 unites (*n* = 6); ^a–d^ Means within a row with no common superscripts differ significantly (*p* < 0.01).

**Table 4:** Se concentration (mg/kg) and total selenium amount (mg) in egg albumen, yolk, and whole egg from laying hens fed DHA-enriched diets with sodium selenite or selenium yeast supplementation.

Item	CON	MA	MA + SS	MA + SY	*p*-Value
Se concentration					
Albumen	0.061 ± 0.002 ^c^	0.059 ± 0.004 ^c^	0.110 ± 0.014 ^b^	0.239 ± 0.010 ^a^	<0.001
Yolk	0.228 ± 0.017 ^b^	0.216 ± 0.016 ^b^	0.603± 0.035 ^a^	0.598± 0.038 ^a^	<0.001
Total Se amount					
Albumen	2.44 ± 0.09 ^c^	2.40 ± 0.20 ^c^	4.33 ± 0.62 ^b^	9.61 ± 0.36 ^a^	<0.001
Yolk	3.46 ± 0.29 ^b^	3.33 ± 0.25 ^b^	9.15 ± 0.60 ^a^	9.21 ± 0.39 ^a^	<0.001
Whole egg	5.90 ± 0.23 ^c^	5.73 ± 0.24 ^c^	13.48 ± 0.91 ^b^	18.82 ± 0.31 ^a^	<0.001

Total selenium amount in whole egg (sum of albumen and yolk Se, excluding eggshell). ^a–c^ Means within a row with no common superscripts differ significantly (*p* < 0.01).

**Table 5:** Chemical composition of table eggs from laying hens fed DHA-enriched diets with sodium selenite or selenium yeast supplementation.

Item	CON	MA	MA + SS	MA + SY	*p*-Value
Yolk composition					
Dry matter, %	52.09 ± 1.00	52.55 ± 0.83	53.13 ± 0.43	52.92 ± 0.97	0.181
Ash, %	1.98 ± 0.20	2.01 ± 0.18	2.08 ± 0.13	2.11 ± 0.13	0.502
Total lipid, %	35.86 ± 2.64	34.56 ± 1.56	34.89 ± 2.16	34.64 ± 1.45	0.673
Proteins, %	16.28 ± 1.10	16.61 ± 1.27	15.94 ± 1.08	16.57 ± 1.41	0.764
Total cholesterol, mg/g egg	13.24 ± 1.59	13.49 ± 1.52	13.07± 2.00	12.55 ± 1.63	0.804
DHA, mg/100 g yolk	65.17 ± 2.42 ^c^	219.89 ± 8.90 ^b^	222.56 ±7.41 ^ab^	233.78 ± 8.14 ^a^	<0.001
Albumen composition					
Dry matter, %	14.66 ± 0.50	14.54 ± 0.40	14.50 ± 0.41	14.61 ± 0.60	0.658
Ash, %	0.74 ± 0.05	0.69 ± 0.05	0.72 ± 0.04	0.71 ± 0.03	0.460
Proteins, %	13.80 ± 0.38	13.69 ± 0.56	13.47 ± 0.32	13.62 ± 0.25	0.453

Each value represents the mean of 6 unites (*n* = 6); ^a–c^ Means within a row with no common superscripts differ highly significantly (*p* < 0.01).

**Table 6:** Oxidative stability of albumen and yolk during storage at room temperature for 0, 15 and 30 days.

Item	CON	MA	MA + SS	MA + SY
Albumen GPX activity, U/g protein				
0 days	69.42 ± 7.67 ^b,x^	65.70 ± 4.34 ^b,x^	88.30 ± 5.25 ^a,x^	91.84 ± 3.72 ^a,x^
15 days	57.49 ± 4.47 ^b,y^	57.75 ± 6.24 ^b,y^	64.82 ± 6.11 ^ab,y^	73.26 ± 7.34 ^a,y^
30 days	51.59 ± 3.35 ^bc,y^	47.12 ± 2.82 ^c,z^	57.78 ± 4.23 ^ab,y^	64.54 ± 7.36 ^a,y^
Albumen carbonyl content, nm/mg protein				
0 days	4.44 ± 0.74 ^ab,z^	5.38 ± 0.49 ^a,z^	4.51 ± 0.41 ^ab,z^	4.40 ± 0.62 ^b,z^
15 days	6.87 ± 0.71 ^ab,y^	7.73 ± 0.77 ^a,y^	6.20 ± 0.40 ^bc,y^	5.61 ± 0.46 ^c,y^
30 days	8.40 ± 0.63 ^a,x^	9.19 ± 0.50 ^a,x^	7.31 ± 0.31 ^b,x^	6.61 ± 0.88 ^b,x^
Yolk GPX activity, U/g protein				
0 days	53.59 ± 4.99 ^b,x^	50.75 ± 5.69 ^b,x^	64.63 ± 6.06 ^a,x^	71.71 ± 6.53 ^a,x^
15 days	49.93 ± 4.48 ^b,xy^	47.07 ± 5.22 ^b,x^	59.62 ± 4.60 ^a,x^	66.33 ± 4.58 ^a,x^
30 days	41.25 ± 6.21 ^b,y^	38.85 ± 2.97 ^b,y^	51.80 ± 3.33 ^a,y^	58.10 ± 2.38 ^a,y^
TBARS in yolk, μg MDA/kg				
0 days	166.6 ± 13.3 ^ab,z^	182.1 ± 8.7 ^a,y^	153.5 ± 12.4 ^bc,y^	145.7 ± 9.5 ^c,y^
15 days	353.0 ± 24.8 ^b,y^	438.0 ± 20.9 ^a,x^	337.2 ± 27.5 ^b,x^	317.2 ± 31.1 ^b,x^
30 days	403.3 ± 12.8 ^bc,x^	495.8 ± 26.0 ^a,x^	408.7 ± 42.9 ^b,x^	361.2 ± 20.3 ^c,x^

Values are means ± standard deviations (*n* = 6) where means between diets on the same day with different letters (^a–c^) differ significantly (*p* < 0.05), and means between days within the same diet with different letters (^x–z^) differ significantly (*p* < 0.05).

**Table 7:** Freshness parameters of DHA-enriched eggs during storage at room temperature for 0, 15, and 30 days.

Item	CON	MA	MA + SS	MA + SY
Weight loss rate, %				
15 days	2.37 ± 0.25 ^b,y^	3.12 ± 0.19 ^a,y^	2.77 ± 0.42 ^ab,y^	2.36 ± 0.38 ^b,y^
30 days	4.95 ± 0.35 ^b,x^	5.97 ± 0.63 ^a,x^	5.09 ± 0.44 ^b,x^	4.37 ± 0.41 ^b,x^
Albumen height, mm				
0 days	7.20 ± 0.31 ^x^	7.10 ± 0.47 ^x^	7.13 ± 0.31 ^x^	7.13 ± 0.26 ^x^
15 days	5.15 ± 0.40 ^a,y^	4.40 ± 0.24 ^b,y^	5.00 ± 0.48 ^a,y^	5.20 ± 0.36 ^a,y^
30 days	4.20 ± 0.24 ^a,z^	3.72 ± 0.22 ^b,z^	4.30 ± 0.48 ^a,y^	4.52 ± 0.63 ^a,y^
Haugh unit				
0 days	84.56 ± 2.02 ^x^	83.96 ± 2.79 ^x^	84.36 ± 1.81 ^x^	84.25 ± 1.69 ^x^
15 days	70.04 ± 3.40 ^a,y^	63.54 ± 2.65 ^b,y^	72.19 ± 4.10 ^a,y^	70.60 ± 2.99 ^a,y^
30 days	61.48 ± 2.23 ^a,z^	54.93 ± 2.58 ^b,z^	59.62 ± 5.79 ^a,z^	62.97 ± 5.51 ^a,y^
Yolk pH				
0 days	6.06 ± 0.06 ^z^	6.03 ± 0.10 ^z^	6.06 ± 0.12 ^z^	6.00 ± 0.12 ^y^
15 days	6.48 ± 0.11 ^y^	6.54 ± 0.07 ^y^	6.44 ± 0.07 ^y^	6.40 ± 0.10 ^x^
30 days	6.70 ± 0.10 ^ab,x^	6.82 ± 0.13 ^a,x^	6.53 ± 0.14 ^bc,x^	6.49 ± 0.07 ^c,x^
Yolk index				
0 days	0.50 ± 0.01 ^x^	0.51 ± 0.02 ^x^	0.50 ± 0.01 ^x^	0.51 ± 0.02 ^x^
15 days	0.45 ± 0.02 ^bc,y^	0.44 ± 0.01 ^c,y^	0.46 ± 0.02 ^ab,y^	0.47 ± 0.02 ^a,y^
30 days	0.41 ± 0.01 ^bc,z^	0.39 ± 0.02 ^c,z^	0.42 ± 0.02 ^b,z^	0.44 ± 0.01 ^a,z^

Values are means ± standard deviations (*n* = 6) where means between diets on the same day with different letters (^a–c^) differ significantly (*p* < 0.05), and means between days within the same diet with different letters (^x–z^) differ significantly (*p* < 0.05).

## Data Availability

All data generated or analyzed during this study are included in the manuscript. For any additional information or requests, please contact the corresponding author.
